# Phosphocaveolin-1 Enforces Tumor Growth and Chemoresistance in Rhabdomyosarcoma

**DOI:** 10.1371/journal.pone.0084618

**Published:** 2014-01-10

**Authors:** Fiorella Faggi, Stefania Mitola, Guglielmo Sorci, Francesca Riuzzi, Rosario Donato, Silvia Codenotti, Pietro Luigi Poliani, Manuela Cominelli, Raffaella Vescovi, Stefania Rossi, Stefano Calza, Marina Colombi, Fabio Penna, Paola Costelli, Ilaria Perini, Maurilio Sampaolesi, Eugenio Monti, Alessandro Fanzani

**Affiliations:** 1 Department of Molecular and Translational Medicine, University of Brescia, Brescia, Italy; 2 Department of Experimental Medicine and Biochemical Sciences, University of Perugia, Perugia, Italy; 3 Department of Experimental Medicine and Oncology, University of Torino, Torino, Italy; 4 Stem Cell Research Institute, University Hospital Gasthuisberg, Leuven, Belgium; 5 Human Anatomy Section, University of Pavia, Pavia, Italy; 6 Interuniversity Institute of Myology (IIM), Italy; University of Kentucky College of Medicine, United States of America

## Abstract

Caveolin-1 (Cav-1) can ambiguously behave as either tumor suppressor or oncogene depending on its phosphorylation state and the type of cancer. In this study we show that Cav-1 was phosphorylated on tyrosine 14 (pCav-1) by Src-kinase family members in various human cell lines and primary mouse cultures of rhabdomyosarcoma (RMS), the most frequent soft-tissue sarcoma affecting childhood. Cav-1 overexpression in the human embryonal RD or alveolar RH30 cells yielded increased pCav-1 levels and reinforced the phosphorylation state of either ERK or AKT kinase, respectively, in turn enhancing *in vitro* cell proliferation, migration, invasiveness and chemoresistance. In contrast, reducing the pCav-1 levels by administration of a Src-kinase inhibitor or through targeted Cav-1 silencing counteracted the malignant *in vitro* phenotype of RMS cells. Consistent with these results, xenotransplantation of Cav-1 overexpressing RD cells into nude mice resulted in substantial tumor growth in comparison to control cells. Taken together, these data point to pCav-1 as an important and therapeutically valuable target for overcoming the progression and multidrug resistance of RMS.

## Introduction

Caveolins (i.e. Cav-1, Cav-2 and Cav-3) are 21–24 kDa membrane-associated proteins that mainly localize in the 50–100 nm cholesterol-enriched invaginations of the plasma membrane, known as *caveolae* [1]–[3]. Caveolins are pivotally involved in multiple processes, including endocytosis, cholesterol homeostasis, regulation of signal transduction and *caveolae* biogenesis that requires the complementary action of Cavin family members [4]–[6]. Cav-1, in particular, has been shown to mostly inhibit a large number of signaling pathways because of the presence of a caveolin scaffolding domain that allows the binding of a plethora of proteins, such as epidermal growth factor receptor, protein kinases C, endothelial nitric oxide synthase [7]. In response to a variety of stimuli such as growth factors, UV irradiation, mechanical and oxidative stress, Cav-1 can also be phosphorylated on tyrosine 14 (hereafter referred as to pCav-1) by members of the sarcoma kinases (Src-kinases) [8]–[10], in turn leading to activation of pathways linked to cell death or survival [11]. In cancer, there is mounting evidence that pCav-1 occurrence often predicts unfavorable outcome by supporting anchorage-independent cell growth, migration, invasiveness and multidrug resistance [11]–[15]. Rhabdomyosarcoma (RMS) is a pediatric soft-tissue cancer [16]–[20] that arises from various muscle and non-muscle progenitors characterized by interrupted myogenesis [21]–[28]. The current classification includes two major histological variants, known as embryonal (ERMS) and alveolar (ARMS), with the former characterized by a complex genomic aetiogenesis [16], [17] and the latter by the prevalent expression of chimeric transcription factors generated by the fusion of the paired box 3 or 7 with forkhead box O1 (Pax3-Foxo1 or Pax7-Foxo1) as a result of specific chromosomal translocations [29], [30].

Previously we have shown that Cav-1 is consistently expressed in both the histological RMS variants [31], [32]. Here, we provide further evidence that Cav-1 is consistently phosphorylated through a Src-dependent mechanism in various ERMS and ARMS cell lines, playing a pivotal role in tumor growth and chemoresistance.

## Results

### Cav-1 is phosphorylated through a Src-dependent mechanism in RMS cells

**Figure 1:**
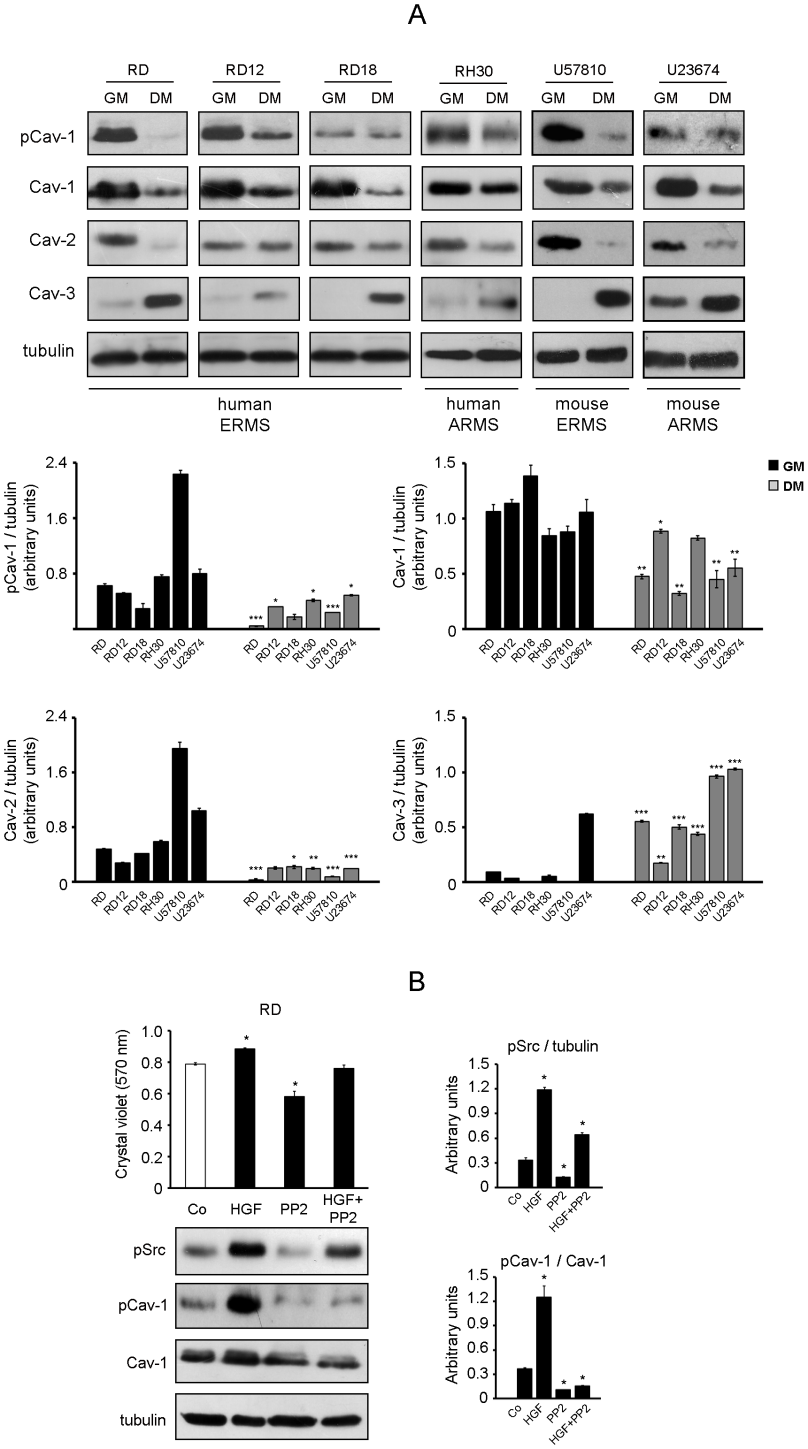
Expression analysis of Caveolins in RMS cells. A) Caveolin protein levels were investigated using human cell lines and mouse primary cultures. Cells were seeded in 60-mm dishes (at a density of 12×10^4^) and cultured in GM until confluence, followed by incubation in DM. After 72 hours in GM or DM, cells were harvested and protein homogenates western blotted for pCav-1, Cav-1, Cav-2, Cav-3 and tubulin. Protein bands were quantified by densitometry after normalization with respect to tubulin (n = 3). *, P<0.05; **, P<0.001; ***, P<0.0001. B) Human RD cells were seeded in 60-mm dishes (at a density of 15×10^4^). After 24 hours, cells were treated or not with 10 ng/ml HGF for 24 hours, in the absence or presence of pre-administered 10 µM PP2. Cells were then harvested and protein homogenates western blotted for pSrc, pCav-1, Cav-1 and tubulin. Protein bands were quantified by densitometry after normalization with respect to tubulin (n = 3). *, P<0.05. After 48 hours, cell proliferation was measured by Crystal violet assay. Histograms represent means ± SD of absorbance. (n = 4) *, P<0.05.

The expression levels of Caveolin family members were analysed by western blot using four human RMS cell lines (embryonal RD, RD12, RD18 and alveolar RH30) and two mouse primary tumor cultures established from transgenic Myf6Cre/p53^−/−^ and Myf6Cre/Pax3-Foxo1/p53^−/−^ mice (embryonal U57810 and alveolar U23674, respectively) [21], [24]. In cells maintained in a growth medium (GM) we observed co-expression of Cav-1 (both Tyr14-phosphorylated and total forms) with Cav-2 and lack or very low expression of Cav-3 (Fig. 1A). Instead, treatment of cells with a differentiation medium (DM) lead to down-regulation of both Cav-1 and Cav-2 and increased Cav-3 levels (Fig. 1A). It is well established that Cav-1 is a substrate of Src-kinase family members [8]-[10], which are upstream activated by different tyrosine kinase receptors involved in cell proliferation and survival upon binding with ligands such as hepatocyte growth factor (HGF), platelet-derived growth factor, insulin and insulin-like growth factor [33]–[37]. Thus, treatment of RD cells with HGF, a growth factor playing a pivotal role in RMS progression [38]–[40], elicited increasing pSrc and pCav-1 levels compared to untreated cells (Fig. 1B, western blot), in turn promoting a rise in cell proliferation (Fig. 1B, Crystal violet assay). In contrast, the effects promoted by HGF were counteracted by co-treatment with a synthetic Src-kinase inhibitor, known as PP2 (Fig. 1B), and similar results were obtained in mouse cultures (not shown). These data point to pCav-1 as a downstream target of Src-kinases especially during proliferation of RMS cells.

### pCav-1 levels affect cell proliferation

**Figure 2:**
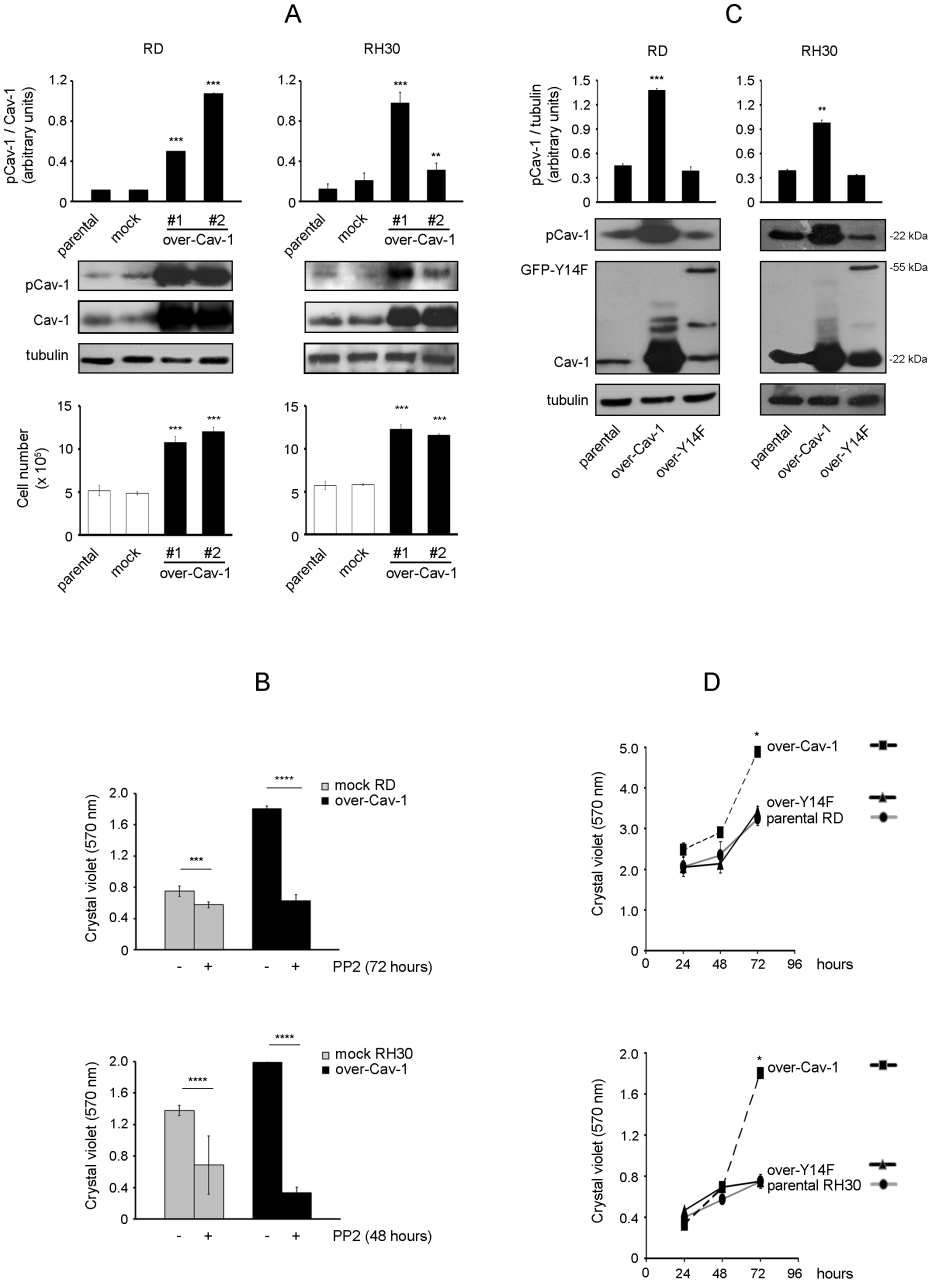
Effects of Cav-1 overexpression on cell proliferation. RD and RH30 cells were stably transfected with either Cav-1 construct (over-Cav-1) or empty vector (mock). A) Parental cells and selected clones were seeded in 60-mm dishes (at a density of 15×10^4^). After 48 hours, cells were harvested and protein homogenates western blotted for pCav-1, Cav-1 and tubulin. Protein bands were quantified by densitometry after normalization with respect to tubulin (n = 3). **, P<0.001; ***, P<0.0001. At the same time points, cell proliferation was evaluated with Burker chamber, as shown in the bottom graphs. Bar graphs represent means ± SD of total cell numbers. (n = 3) ***, P<0.0001. B) Mock and over-Cav-1 cells were seeded in 24-well plates (at a density of 15×10^3^). After 24 hours, cells were either treated with dimethylsulfoxide (DMSO, vehicle) or 10 µM PP2 (replenished every 24 or 16 hours for RD and RH30 cells, respectively) for the indicated time points. Cell proliferation was then evaluated by Crystal violet assay. Histograms represent means ± SD of absorbance (n = 4). ***, P<0.0001; ****, P<2e-16. C) RD and RH30 cells were stably transfected with constructs for either Cav-1 (22 kDa) or non-phosphorylatable GFP-Y14F (55 kDa). Parental cells and selected clones were seeded in 60-mm dishes (at a density of 12×10^4^). After 24 hours, cells were harvested and protein homogenates western blotted for pCav-1, Cav-1 and tubulin. Protein bands were quantified by densitometry after normalization with respect to tubulin (n = 3). **, P<0.001; ***, P<0.0001. D) Under the same conditions seen above, cell proliferation was evaluated by Crystal violet assay at the indicated time points. Histograms represent means ± SD of absorbance (n = 4). *, P<0.05.

**Figure 3:**
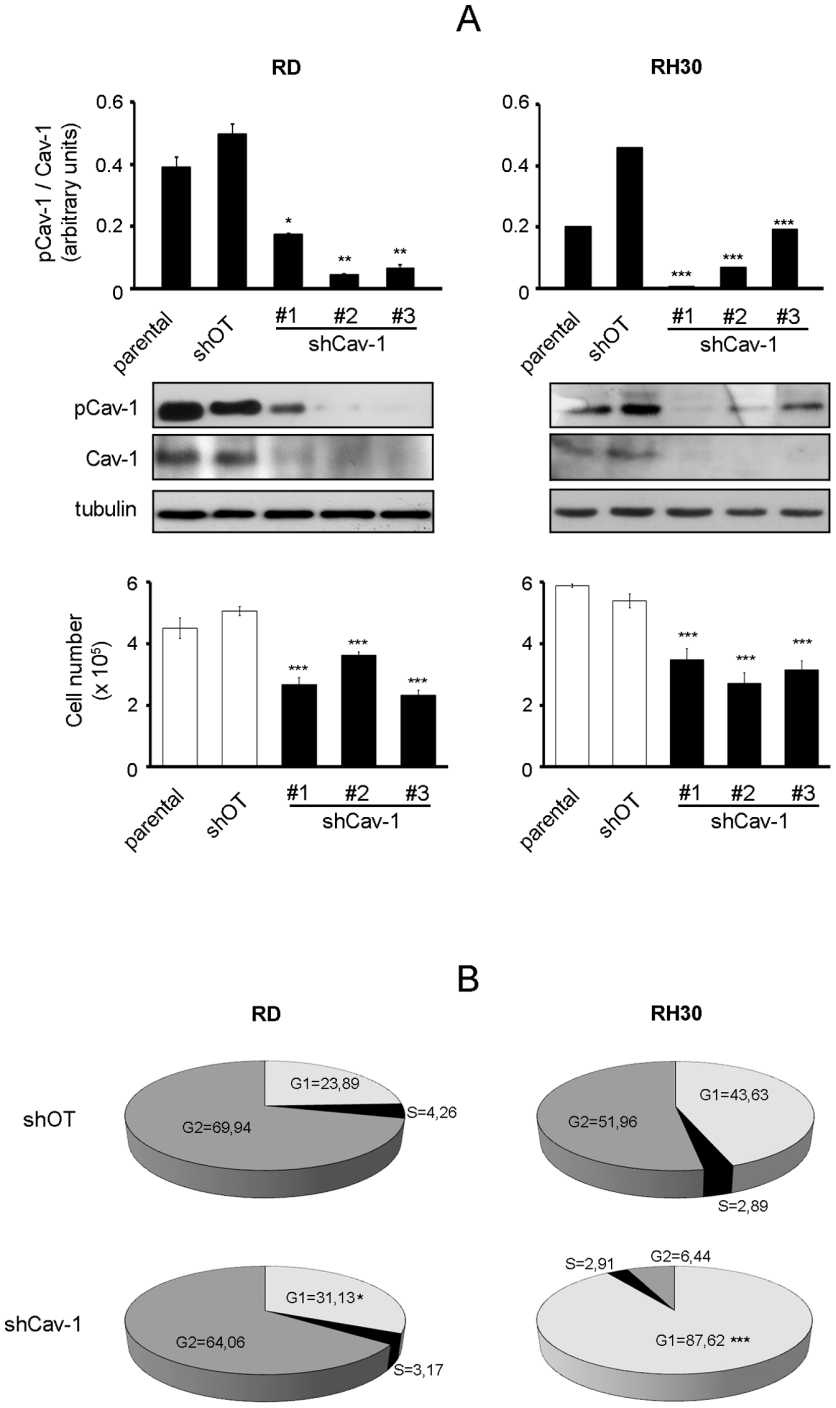
Effects of Cav-1 knockdown on cell proliferation. RD and RH30 cells were stably transfected with either Cav-1 knockdown (shCav-1) or off-target (shOT) construct. A) Parental cells and selected clones were seeded in 60-mm dishes (at a density of 12×10^4^). After 48 hours, cells were harvested and protein homogenates western blotted for pCav-1, Cav-1 and tubulin. Protein bands were quantified by densitometry after normalization with respect to tubulin (n = 3). *, P<0.05; **, P<0.001; ***, P<0.0001. At the same time points, cell proliferation was evaluated with Burker chamber, as shown in the bottom graphs. Bar graphs represent means ± SD of total cell numbers (n = 3). ***, P<0.0001. B) FACS analysis was performed on shCav-1 and shOT cells. Numbers in pie charts denote percent cells in the G1/S/G2-phases of the cell cycle (n = 3). *, P<0.05; ***, P<0.0001.

To gain further insights into the role of pCav-1, we investigated the effects of Cav-1 overexpression and knockdown using the human RD and RH30 cell lines, which are widely employed as particularly representative of ERMS and ARMS histotypes, respectively [41]. As observed in two distinct clones of each cell line, Cav-1 overexpression correlated with increased pCav-1 levels (Fig. 2A, western blot), leading to a significantly faster proliferation compared to controls, as evaluated by cell counting (Fig. 2A, bottom graph). Consistently, PP2 treatment was sufficient to override the rise in cell proliferation induced by Cav-1 overexpression and to inhibit the proliferation of controls as well (Fig. 2B). In addition, cells stably transfected with a green fluorescent protein-tagged non-phosphorylatable Cav-1 form (GFP-Y14F), having pCav-1 levels comparable to parental cells (Fig. 2C), exhibited a normal cell proliferation (Fig. 2D). On the other side, the loss of pCav-1 obtained by Cav-1 knockdown in both the cell lines (Fig. 3A, western blot) determined an impaired proliferative ability, as observed by cell counting (Fig. 3A, bottom graph). In particular, Cav-1 knockdown induced an increase in G1 phase compared to controls (Fig. 3B), indicating a link between Cav-1 and the cell cycle machinery.

### pCav-1 levels impact the phosphorylation state of ERK and AKT kinases

**Figure 4:**
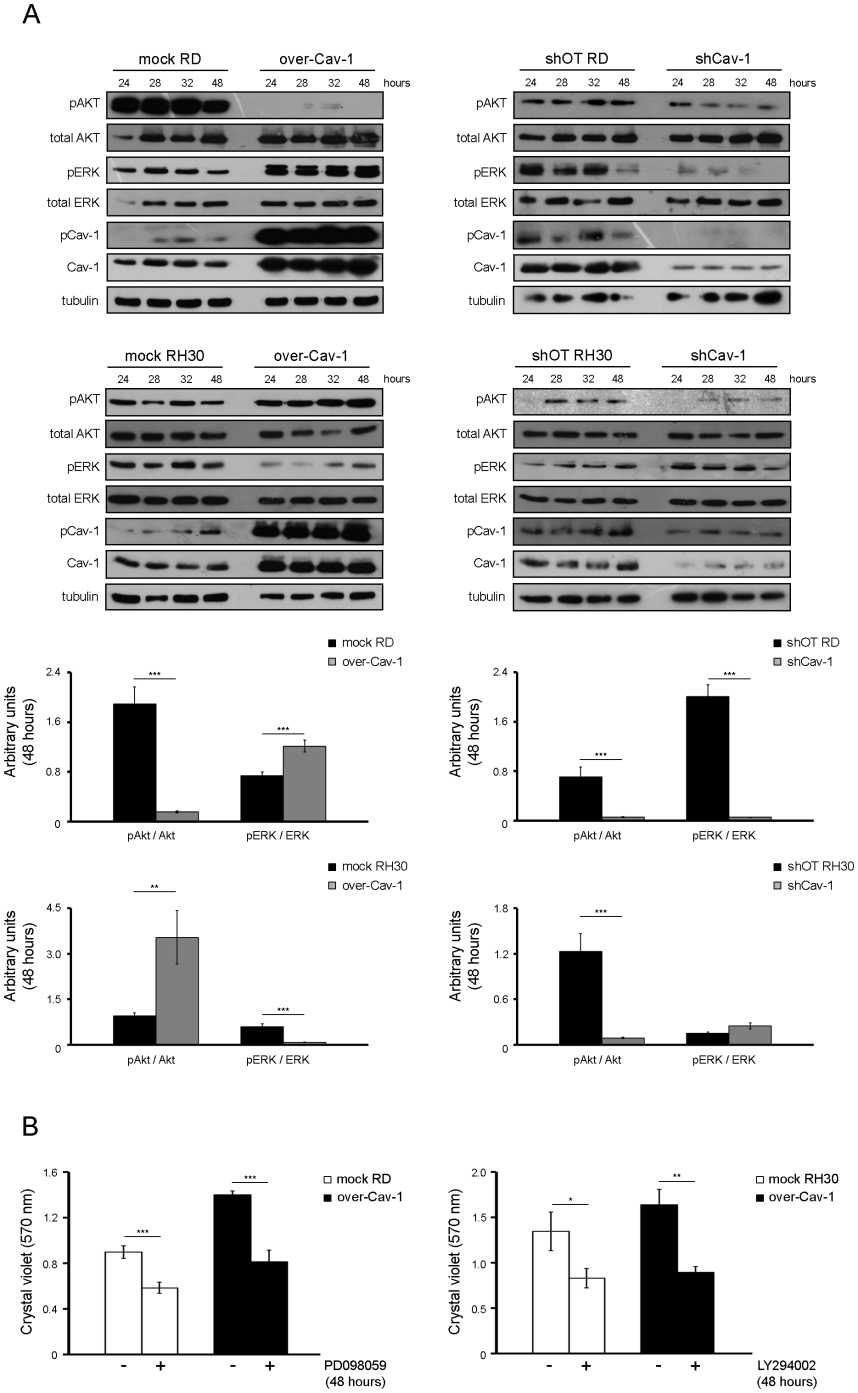
Effects of Cav-1 levels on the phosphorylation state of ERK and AKT. Control, over-Cav-1 cells and shCav-1 cells were seeded in 60-mm dishes (at a density of 12×10^4^). A) At the indicated time points, cells were harvested and protein homogenates western blotted for pAKT, AKT, pERK, ERK, pCav-1, Cav-1 and tubulin at the indicated time points. Protein bands corresponding to pAKT and pERK levels (as detected at 48 hours) were quantified by densitometry after normalization with respect to tubulin (n = 3). **, P<0.001; ***, P<0.0001. B) 24 hours after seeding, mock and over-Cav-1 cells were treated with 15 µM PD09859 (in the case of RD cell line) or 10 µM LY294002 (in the case of RH30 cell line) for up to 24 hours. Cell proliferation was then evaluated by Crystal violet assay. Histograms represent means ± SD of absorbance (n = 4). *, P<0.05; **, P<0.001; ***, P<0.0001.

We evaluated whether pCav-1 could impact the activation of protein kinase B (known as AKT) and the extracellular regulated kinases (ERK), two critical regulators of cell proliferation and survival in several malignancies [42]. In the RD or RH30 cells, Cav-1 overexpression specifically enhanced the phosphorylation state of either ERK or AKT, respectively, as compared to controls (Fig. 4A, left panels). In addition, the reinforcement of each specific pathway was characterized by the inhibition of the other one (Fig. 4A, left panels). We were also able to verify that the augmented cell proliferation due to Cav-1 overexpression was consistently overwhelmed by treatment with PD098059 in RD cells or with LY294002 in RH30 cells, two compounds inhibiting the ERK and AKT pathways, respectively (Fig. 4B). On the other side, Cav-1 knockdown determined a down-regulation of pERK and pAKT levels in both the cell lines, though at later time points in RH30 cells (Fig. 4A, right panels), thus suggesting that gain or loss of Cav-1 may impact the activation state of ERK and AKT kinases in a cell-context dependent manner.

### pCav-1 promotes cell migration and invasion *in vitro* and tumor growth *in vivo*

**Figure 5:**
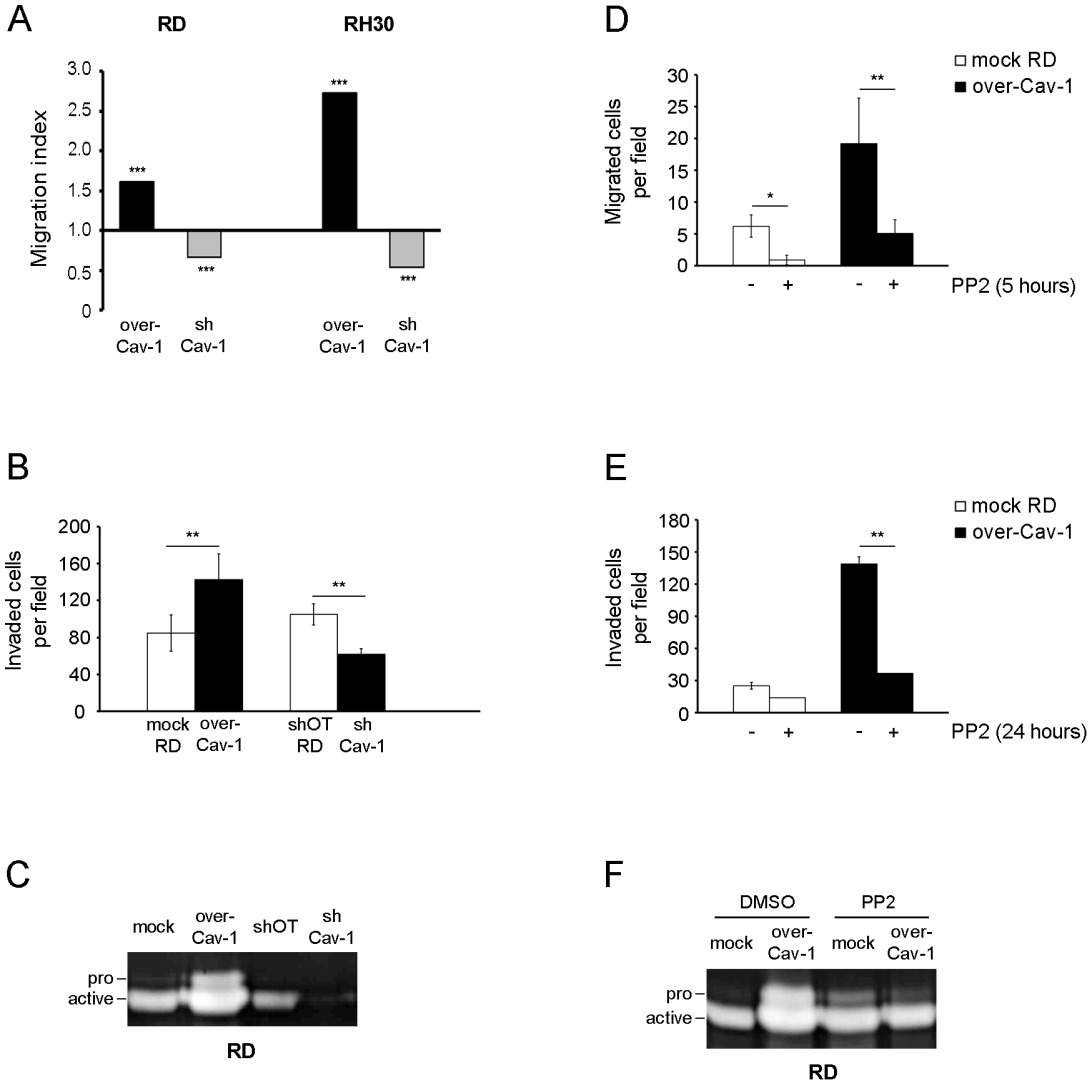
Effects of Cav-1 levels on cell migration and invasiveness. A) Cell migration was evaluated using a modified Boyden chamber assay. The migration index was calculated through the ratio between the mean number of migrated clones (over-Cav-1 and shCav-1) with respect to their migrated controls, as counted in five randomly chosen fields (n = 3). ***, P<0.0001. B) Cell invasiveness of RD clones (over-Cav-1 and shCav-1) in comparison to controls was evaluated by Matrigel assay. Histograms represent mean ± SD (n = 3). **, P<0.001. C) Gelatin-zymography assay was performed to evaluate the MMP2 activation in RD clones (over-Cav-1 and shCav-1) with respect to controls. D-E-F) Cell migration (D), invasiveness (E) and the MMP2 activation (F) were evaluated in RD clones (mock and over-Cav-1) treated or not with 10 µM PP2 for 24 hours. Histograms represent means ± SD (n = 3). *, P<0.05; **, P<0.001.

**Figure 6:**
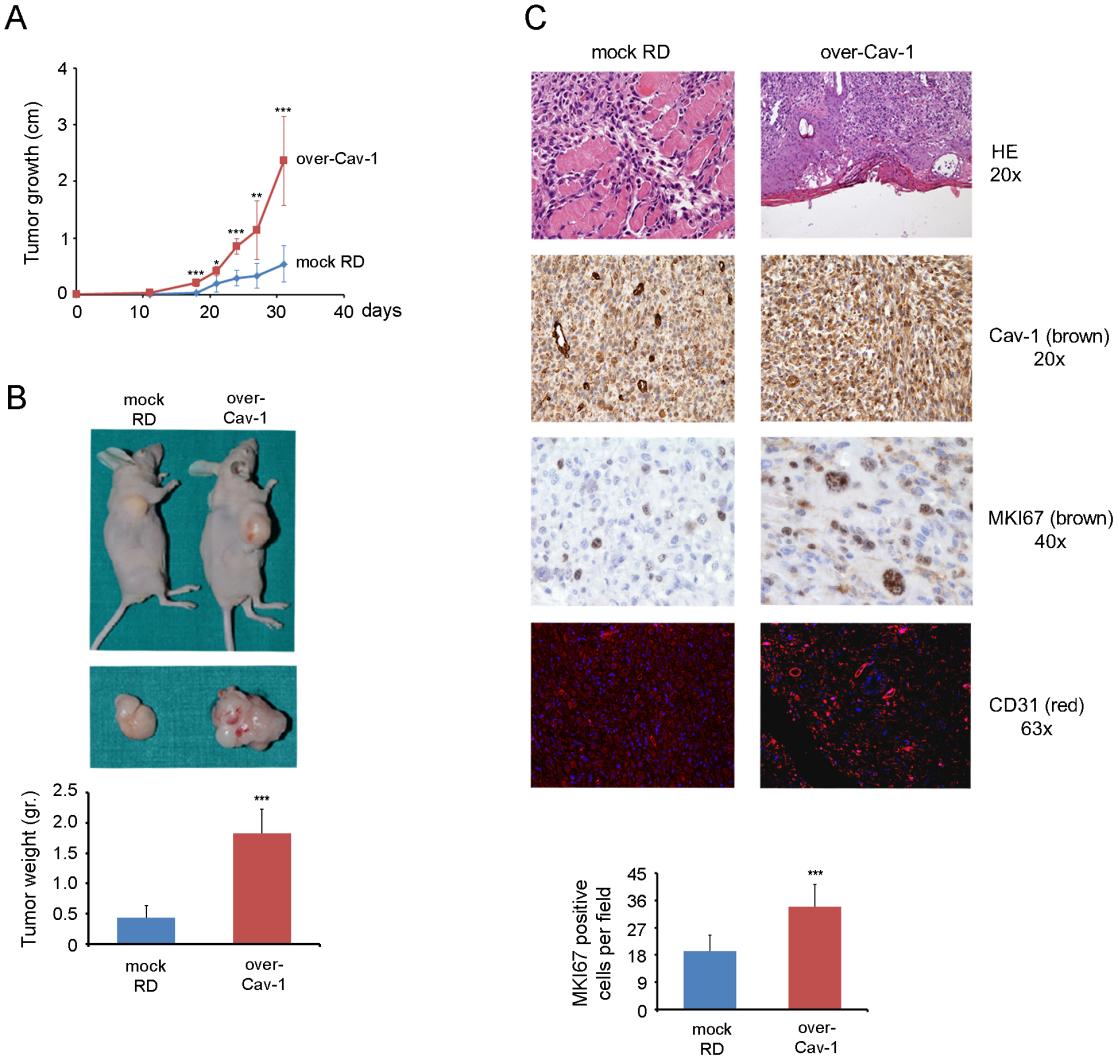
Effects of Cav-1 overexpression on tumor growth *in vivo*. A) Mock and over-Cav-1 RD clones (35×10^5^ cells) were subcutaneously injected into the back of nude mice (n = 8). The size of tumors was measured six times during the subcutaneous growth. Histograms represent means ± SD of tumor weight. *, P<0.05; **, P<0.001; ***, P<0.0001. B) After 31 days mice were sacrificed and the weight of collected tumors was measured. ***, P<0.0001. C) Tumor sections were stained with Hematoxilin and Eosin (HE) and immunostained for Cav-1, MKI67 and CD31. Quantification of MKI67 is reported in the bottom graph. Histograms represent means ± SD of MKI67 positive cells as counted in five randomly chosen fields. (n = 3). ***, P<0.0001.

By employing a Boyden chamber assay we observed an increased or reduced migration of RD and RH30 cells upon Cav-1 overexpression and silencing compared to controls, respectively (Fig. 5A). Likewise, the increased or decreased Cav-1 levels correlated with the degree of cell invasiveness and activation of the matrix metalloprotease-2 (MMP2) [43], as observed by means of Matrigel and zymography assays performed on RD cells (Fig. 5B and 5C, respectively). In addition, PP2 treatment was sufficient to overwhelm the increased migration (Fig. 5D), invasion (Fig. 5E) and MMP2 activation of Cav-1 overexpressing RD cells (Fig. 5F). We then evaluated the effects of Cav-1 overexpression on tumor growth *in vivo*. To this purpose, mock and Cav-1 overexpressing RD cells were subcutaneously injected into the back of nude mice (n = 8). Tumor growth was significantly accelerated in mice injected with Cav-1 overexpressing RD cells (Fig. 6A), yielding huge tumor masses characterized by a visible increased vascularization and a weight of about 4 times higher compared to controls (Fig. 6B). As determined by immunohistochemistry, the bigger tumor masses with expectedly high Cav-1 expression were characterized by increased staining with marker of proliferation Ki-67 (MKI67, also known as MIB-1) and cluster of differentiation 31 (CD31), two markers predictive of cell mitosis and vascularization, respectively (Fig. 6C). Of note, we were unable to assess whether the loss of Cav-1 could lead to a regression of tumor growth *in vivo*, because the injected shCav-1 cells had lost the silencing effects yielding tumor masses largely positive for Cav-1 (not shown).

### pCav-1 contributes to chemoresistance

**Figure 7:**
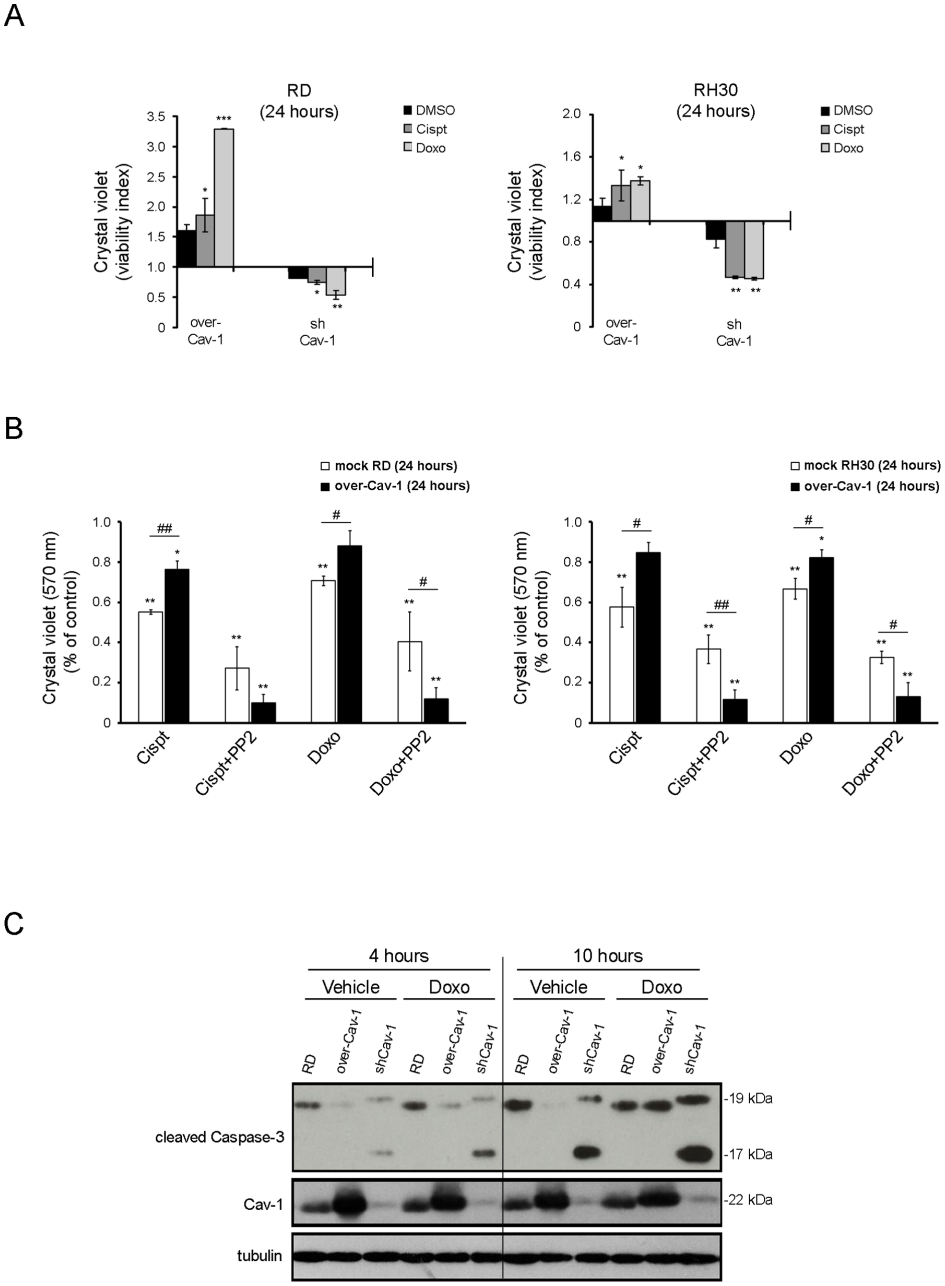
Effects of Cav-1 levels on chemoresistance *in vitro*. A) Cells were seeded into a 24 well plate (at a density of 15×10^3^). After 24 hours, cells were treated with cisplatin (0.2 ng/ml) or doxorubicin (2 µg/ml) and DMSO as control for up to 24 hours. Crystal violet assay was then performed and the viability index was calculated by dividing the mean number of viable cells in Cav-1 overexpressing or silenced clones through their respective controls Histograms represent means ± SD of absorbance (n = 4). B) Cristal violet assay was carried out to evaluate the percentage of viable Cav-1 overexpressing and control cells after treatment with either cisplatin or doxorubicin in the absence or presence of 10 µM PP2 for 24 hours. Histograms represent means ± SD of absorbance (n = 4). *, P<0.05; **, P<0.001; **, P<0.0001 versus untreated cells. #, P<0.05; ##, P<0.001. C) Cleavage of Caspase-3 was evaluated by western blot analysis in over-Cav-1, shCav-1 RD clones and parental cells treated or not with doxorubicin for the indicated time points.

Since Cav-1 is long known to play a pivotal role in chemoresistance [44], the cell survival of Cav-1 clones was tested in the presence of chemotherapeutic compounds. In comparison to control cells, Cav-1 overexpressing and knockdown cells were respectively more resistant or sensitized to cell death in response to cisplatin or doxorubicin treatment, as deduced by Crystal violet assay (Fig. 7A). Significantly, the drug resistance of Cav-1 overexpressing cells was almost abolished by co-treatment with PP2 (Fig. 7B), suggesting the contribute of pCav-1 to multidrug resistance. In addition, Cav-1 overexpression and knockdown respectively protected or sensitized cells to a caspase-dependent apoptosis [45], as evaluated by western blot analysis of the active caspase-3 fragments (19 and 17 kDa) (Fig. 7C). This set of experiments shows that overexpression or lack of Cav-1 cooperates to protect from or enhance a caspase-dependent apoptosis in RMS cells, respectively.

## Discussion

*Cav-1* and *Cav-2* genes are located in a fragile site (known as D7S522 locus) that is frequently deleted in human cancers [46], indicating a common role as tumor suppressors. Consistent with these observations, an impaired Cav-1 expression was found in human lung [47], mammary [48], colon [49] and ovarian carcinomas [50] or sarcomas as well [51]. Cav-1 and Cav-2 conventionally form plasma membrane hetero-oligomers that negatively regulate the activity of several receptors involved in cell proliferation and survival [52]–[54], and therefore their loss is thought to facilitate tumor progression by deliberate activation of different signaling pathways, as observed in the tumor prone Cav-1 knock-out mouse model [55]–[59]. Yet, additional mechanisms complicating the scenario have been described, since the inhibitory interaction of Cav-1 with the epidermal growth factor receptor can be hindered by the formation of a galectin-glycoprotein lattice, thereby resulting in sustained receptorial activity, as observed in breast tumor cells [60], [61]. Beyond functioning as a tumor suppressor, accumulating evidence have more recently indicated that Cav-1, especially when phosphorylated in Tyr14, behaves as an ambiguous partner in cancer [11]–[15], because of its ability to activate pathways involved in cell migration and invasion, such as the Rho/ROCK and Focal adhesion kinases signaling systems [62]. The data presented here point to pCav-1 as a positive modulator of proliferation, migration, invasiveness, chemoresistance *in vitro* and tumor growth *in vivo* in RMS, the most frequent childhood soft tissue sarcoma characterized by expression of myogenic markers and impaired differentiation [21]–[28]. The malignant cell phenotype we observed by Cav-1 overexpression was ascribable to the robust increase of Cav-1 phosphorylation in Tyr14, likely due to an increased accessibility of the intracellular Cav-1 pool to Src-family members, as observed in other cellular models [63], [64]. In this regard, further studies are required to examine whether and how Cav-1 overexpression may perturb the *caveolae* organization and Cav-1 trafficking in RMS cells. Among its many features, Cav-1 has widely been reported to exert both inhibitory and activatory functions on the RAS/ERK and AKT pathways depending on the cellular context (see for a review [11]–[15]) and, more recently, pCav1 was found to be specifically interconnected with ERK and AKT signaling in mouse embryonic stem cells [65]. In this context, it is quite of interest that we observed pCav-1 reinforcing the proliferation of the embryonal RD or alveolar RH30 cells via specific increase of either ERK or AKT signaling, respectively, since activating *RAS* mutations leading to sustained ERK signaling are solely detected in ERMS [66]–[70] and the Pax3-Foxo1 factor strongly cooperates with activated AKT signaling in ARMS malignancy [71], [72]. An additional cue of interest was that we observed pCav-1 cooperating to the overactivation of one specific pathway and causally inhibition of the other one (or *viceversa*). It is largely documented that AKT and ERK signaling influence with each other by positive or negative crosstalks in a cell-context dependent manner [73], and a recent report has shown that if one of these two pathways is chemically inhibited in various RMS cell lines, including RD and RH30 cells, they opportunistically potentiate the other one to survive [74]. Intriguingly, the same authors showed that the block of either pathway did not result in the potentiation of the other one in the RMS TE671 cell line [74], in which we have previously shown the lack of Cav-1 expression [32], therefore cautiously suggesting that Cav-1 may participate to the negative ERK/AKT crosstalk observed in some RMS cells.

Finally, we observed such a clear connection between Cav-1 and the cell cycle machinery of RMS cells, as analogously reported for human endothelial cells [75]. Cav-1 knockdown induced an accumulation in G1 phase and subsequent block of cell proliferation characterized by down-regulation of both ERK and AKT. Thus, the loss of Cav-1 could mimic to some extent the pro-apoptotic effects produced by the concomitant chemical ERK/AKT inhibition [74], [76], thereby sensitizing RMS cells to cell death in response to chemotherapeutic agents, definitely pointing to pCav-1 as a therapeutically valuable target to overcome RMS malignancy. In this perspective, since the recent growing body of literature indicating a cooperation between Cav-1 and the polymerase transcription released factor PTRF/Cavin-1 in cancer progression [4], [77]–[85], it will be certainly important to assess their relative contribution in RMS malignancy.

## Materials and Methods

All reagents were from Sigma-Aldrich (Milan, Italy), unless otherwise stated.

### Ethic statement

This study was carried out in strict accordance with the recommendations in the Guide for the Care and Use of Laboratory Animals of the University of Perugia (Italy). The protocol was approved by the Committee on the Ethics of Animal Experiments of the University of Perugia. All surgery was performed under sodium pentobarbital anesthesia, and all efforts were made to minimize suffering.

### Cell culture

Human RD and RH30 cells were purchased from the European Collection of Cell Cultures (ECACC, Salisbury, UK). RD cells harbor *RAS* [86], [87] and *P53* [88] mutations, while RH30 cells express Pax3-Foxo1 [89] and harbor *P53* mutations [88]. RD12 and RD18 cell lines were derived by random cloning of the RD line [90], [91]. The mouse primary cultures, namely U57810 (ERMS) and U23674 (ARMS), were established from the transgenic Myf6Cre/*p53* ^−/−^ and Myf6Cre/*Pax3-Foxo1*/*p53^−/−^* mice, respectively [21], [24]. Cells were routinely maintained under standard conditions (37°C and 5% CO_2_ in humidified incubator) in GM, composed by high-glucose Dulbecco's modified eagle's medium (DMEM) supplemented with 10% foetal bovine serum (FBS) in the presence of 100 µg/ml penicillin/streptomycin antibiotics and 1% L-Glutamine (only for RH30 cells). To induce myodifferentiation, 80% confluent cells were switched to DM, composed by DMEM supplemented with 2% horse serum. Cells were treated with: HGF (10 ng/ml, ImmunoTools, Friesoythe, Germany), a Src-kinase inhibitor known as PP2 (10 µM), the chemotherapeutic drugs doxorubicin (0.15 ng/ml) and cisplatin (2 µg/ml), the synthetic inhibitors of the RAS/ERK and AKT cascades, respectively known as PD098059 (15 µM) and LY294002 (10 µM).

### Plasmids and transfection

The overexpression of either the wild-type or non-phosphorylatable Cav-1 was carried out by transfection of pCAGGS/Cav-1 (provided by F. Galbiati, University of Pittsburgh, USA) or pEGFPN1/Cav-1(Y14F) (provided by D. Maggi, University of Genova, Italy) constructs, respectively. Knockdown constructs were purchased from Sigma-Aldrich in pLKO.1 backbone: shCav-1 (clone TRCN0000011218), 5′-CCGGGACCCACTCTTTGAAGCTGTTCTCGAGAACAGCTTCAAAGAGTGGGTCTTT-3′. As negative controls, pLKO.1-puro harboring the following off-target sequence was used: 5′-CCGGCAACAAGATGAAGAGCACCAACTCGAGTTGGTGCTCTTCATCTTGTTGTTTTT-3′. Cells were stably transfected using Lipofectamine 2000 reagent (Invitrogen) according to the manufacturer's protocol. After antibiotic selection, the experiments raised similar results in all the selected clones.

### Antibodies

Antibodies used for immunoblotting/immunohistochemistry were phospho-Cav-1 (Tyr14) (code 61338, BD Biosciences, Buccinasco, Italy), Cav-1 (code SC-894, Santa Cruz Biotechnology, Dallas, USA), Cav-2 and Cav-3 (code 610684 and 610420, BD Biosciences, Buccinasco, Italy), phosho-Src (Tyr418) (code 569732, Merck-Millipore, Milan, Italy), total and phospho-AKT (Ser473) (code #2920 and #4060, Cell Signalling, Milan, Italy), total and phospho-ERK (Tyr204) (code SC-135900 and SC-7883, Santa Cruz Biotechnology, Dallas, USA), MKI67 (code NCL-L-Ki67-MM1, NovocastraTM Laboratories Ltd, Newcastle Upon Tyne, United Kingdom), CD31 (code SC-8306, Santa Cruz Biotechnology, Dallas, USA), cleaved Caspase-3 (code #9661, Cell Signalling, Milan, Italy) and tubulin (code T5168, Sigma-Aldrich, Milan, Italy).

### Immunoblotting

Protein concentration was calculated by Bradford reagent assay. Equal amounts of protein samples were separated by SDS-PAGE under reducing conditions and transferred to polyvinylidine fluoride membranes. Incubation with specific primary antibodies was followed by horseradish peroxidase-conjugated secondary antibodies (anti-mouse IgG from Santa Cruz Biotechnology, Dallas, USA; anti-rabbit IgG from Thermo Scientific, Erembodegem, Belgium) and the resulting immunocomplexes were visualized using enhanced chemiluminescence reagent (GeneSpin, Milan, Italy). Immunoreactive bands were quantified using densitometric analyses (Software Gel Pro Analyzer, version 4). Total protein homogenates were obtained by harvesting cells in a cold RIPA lysis buffer, composed by 20 mM Tris-HCl (pH 7.6), 1% Nonidet P40, 0.5% sodium deoxycholate, 0.1% SDS, 50 mM NaCl and a cocktail of protease inhibitors (Roche, Milan, Italy) plus phosphatase inhibitors (1 mM Na_3_VO_4_ and 4 mM NaF). For Caveolin analysis, the Triton-insoluble membranous fractions were obtained by centrifugation of cells harvested in a cold Triton buffer, composed by 10 mM Tris-HCl (pH 8.0), 1% Triton X-100, 5 mM EDTA, 150 mM NaCl, and a cocktail of protease and phosphatase inhibitors (15,000×g for 15 minutes at 4°C).

### Cell proliferation assay

Cells were seeded in 60-mm dishes (at a density of 15×10^4^). After 24, 48 and 72 hours, cells were detached and counted with a Burker chamber (in triplicate) by a phase contrast microscope. Alternatively, cells were seeded into a 24 well plate (at a density of 15×10^3^). After 24, 48 or 72 hours, cells were harvested, paraformaldehyde fixed and stained with Crystal violet (0.5% in PBS with 20% methanol). Absorbance was then measured by reading the plate at 570 nm emission wavelength. Images of cell proliferation assays reflect representative results of at least four independent experiments.

### Cell cycle analysis

Cells were seeded in 100-mm dishes (at a density of 70×10^4^). After 24 hours, cells were harvested, fixed in 1 ml of ice-cold methanol and stained with 0.5 ml of propidium iodide (100 µg/ml) containing 5 µg/ml pancreatic RNase (Agilent Technologies, Wilmington, USA) overnight at 4°C. After gating out cellular aggregates and debris, propidium iodide fluorescence was measured using a CyFlowPartec flow cytometer (Partec Italia, Milan, Italy). Data were analysed with FlowJo software (Tree Star, Ashland, USA).

### Chemotaxis assay

Cells (25×10^3^ cells in 50 µl of DMEM with 5% FBS) were seeded in the upper compartment of a Boyden chamber, containing gelatin-coated PVP-free polycarbonate filters (8 µm pore size, Costar, Cambridge, USA) and DMEM with 10% FBS in the lower compartment. As a negative control, 1% FBS medium was used. After 5 hours of incubation at 37°C, cells migrated to the lower side of the filter were stained with Diff-Quik (Dade-Behring, Milan, Italy). Five random fields were counted for each triplicate sample.

### Matrigel assay

Matrigel assay (BD Biosciences, Buccinasco, Italy) was used to assess the cell invasive potential, according to the manufacturer's protocol. Briefly, cells were plated into upper inserts of 24-well plates (at a density of 60×10^3^) and incubated at 37°C. Bottom chambers were filled with 500 µl of DMEM supplemented with 10% FBS as a chemoattractant or DMEM only as a negative control. After 24 hours, non-invading cells were gently removed from the upper surface of inserts with a cotton swab and invaded cells were methanol fixed and stained with Crystal violet (0.1% in PBS with 20% methanol). The number of cells that invaded the filter was counted using a bright-field microscope. Five randomly selected fields were counted for each filter and experiments were performed in triplicate.

### Zymography assay

The activity of MMP2 was assayed by loading 40 µl of low serum (0.1% FBS) cell conditioned medium into 10% SDS-PAGE containing 1 mg/ml incorporated gelatin substrate. Following electrophoresis, the gels were soaked in 2.5% Triton X-100 to remove SDS and incubated for 24 hours at 37°C in the Collagenase Buffer (50 mM Tris-HCl, 5 mM CaCl_2_, 0.02% Na Azide, 0.005% Brij 35, 1 µM ZnCl_2_) with or without 5 mM EDTA. Clear bands were visualized on the blue background after staining with 0.1% Coomassie blue in 40% ethanol and 10% acetic acid and destained in 30% methanol and 10% acetic acid.

### Xenograft experiments

Athymic nude female mice (nu/nu, Harlan Laboratories, Bresso, Italy) weighing ∼20 gr were subcutaneously injected with 35×10^5^ RD cells in the back. Mice were inspected twice a week and sacrificed by cervical dislocation after 5 weeks. Tumor volume was calculated by the equation: tumor volume = x^2^y/2, where x and y correspond to the width and thickness of the tissue, respectively. Tumor masses were then excised, weighed and fixed with 10% formalin in PBS (2 days at 4°C), extensively washed in PBS, and paraffin-embedded.

### Immunohistochemistry

Immunohistochemistry was performed on paraffin sections according to the manufacturer's protocol. Briefly, sections were de-waxed, re-hydrated and endogenous peroxidase activity blocked by 0.3% H_2_O_2_/methanol for 20 minutes. Heat-induced antigen retrieval was performed using a thermostatic bath in 1 mmol/l EDTA (pH 8.0) or 1 mM Citrate buffer (pH 6.0). Sections were then washed in TBS (pH 7.4) and incubated over-night in TBS/1% bovine serum albumin with the specific primary antibody. Single immunostain has been revealed by ChemMATE EnVision HRP Labelled Polymer system (DAKO, Glostrup, Denmark) or NovoLinkTM Polymer Detection System (NovocastraTM Laboratories Ltd, Newcastle Upon Tyne, United Kingdom) followed by diaminobenzydine as chromogen and Hematoxylin and Eosin as counterstain.

### Statistical analysis

Linear models fitted by ordinary least squares or generalised least squares in case modelling the heteroscedasticity were used. The differences between the groups were analyzed by unpaired Student's t test and One-Way ANOVA test (with Dunnet's post-test), using Prism 4 software for Windows (GraphPad Software, San Diego, USA). Statements of significance were based on a p value of less than 0.05.
